# Sex Differences in Cancer and Cardiotoxicity: Mechanisms, Outcomes, and Clinical Implications Across Solid and Hematological Malignancies

**DOI:** 10.3390/cancers18111677

**Published:** 2026-05-22

**Authors:** Kalliopi Keramida, Marianne C. Aznar, Jutta Bergler-Klein, Giuseppe Boriani, Daniela Cardinale, Susan Dent, Alexandra Drakaki, Jose J. Fuster, Mamas A. Mamas, Tochi Okwuosa, Lydia Scarfo, Peter Van Der Meer, Eric H. Yang, Teresa Lopez-Fernandez

**Affiliations:** 1 Cardiology Department, General Anti-Cancer Oncological Hospital Agios Savvas, 11522 Athens, Greece; 2 Department of Cardiology, Athens University Hospital Attikon, Medical School, National and Kapodistrian University of Athens, 12462 Athens, Greece; 3 Division of Cancer Sciences, School of Medical Sciences, Faculty of Biology, Medicine and Health, University of Manchester, Manchester M13 9PL, UK; marianne.aznar@manchester.ac.uk; 4 Department of Cardiology, Medical University of Vienna, 1090 Vienna, Austria; jutta.bergler-klein@meduniwien.ac.at; 5 Division of Cardiology, Department of Biomedical, Metabolic and Neural Sciences, Modena University Hospital, University of Modena and Reggio Emilia, 41121 Modena, Italy; 6 Cardioncology Unit, European Institute of Oncology, Istituto di Ricovero e Cura a Carattere Scientifico, Via Ripamonti 435, 20141 Milan, Italy; 7 Department of Medicine, University of Rochester, Rochester, NY 14627, USA; 8 Division of Hematology/Oncology, Department of Medicine, David Geffen School of Medicine, University of California, Los Angeles, CA 90095, USA; 9 Centro Nacional de Investigaciones Cardiovasculares (CNIC), 28029 Madrid, Spain; jjfuster@cnic.es; 10 Centro de Investigación Biomédica en Red de Enfermedades Cardiovasculares, 28029 Madrid, Spain; 11 Keele Cardiovascular Research Group, Keele University, Stoke-on-Trent ST5 5BG, UK; 12 Division of Cardiology, Department of Internal Medicine, Rush University Medical Center, Chicago, IL 60612, USA; 13 Università Vita Salute San Raffaele, 20132 Milano, Italy; 14 Strategic Research Program on CLL, IRCCS Ospedale San Raffaele, 20132 Milano, Italy; 15 Department of Cardiology, University Medical Center Groningen, University of Groningen, 9712 Groningen, The Netherlands; 16 UCLA Cardio-Oncology Program, Division of Cardiology, Department of Medicine, University of California at Los Angeles, Los Angeles, CA 90095, USA; 17 Cardio-Oncology Unit, Cardiology Department, IdiPAZ Research Institute, La Paz University Hospital, 28046 Madrid, Spain; 18 Cardio-Oncology Unit, Cardiology Department, Quironsalud University Hospital, 28223 Madrid, Spain

**Keywords:** sex, cardiotoxicity, cancer therapy, radiotherapy, hematological malignancies, precision cardio-oncology

## Abstract

Sex differences influence cancer incidence, treatment response, and susceptibility to cardiovascular toxicity. Males exhibit higher rates and poorer outcomes in most non-sex-specific cancers, while females more frequently experience treatment-related adverse events, including cancer therapy-related cardiac dysfunction. Biological factors such as hormonal status, genetic polymorphisms, immune responses, and pharmacokinetics contribute to these disparities. In cardio-oncology, women—particularly premenopausal or with specific genotypes—may be at increased risk for cardiotoxicity after treatment with anthracyclines, immune checkpoint inhibitors or radiotherapy. Clonal hematopoiesis and certain germline genetic variants such as single nucleotide polymorphisms (e.g., RARG rs2229774, HAS3 rs2232228) are emerging as potential sex-informed biomarkers for predicting cardiotoxicity risk. Despite growing evidence, sex remains insufficiently integrated into clinical trials and guideline development in cardio-oncology. This review highlights the importance of sex-specific surveillance, prevention, and multi-omic risk stratification to advance precision cardio-oncology and support better outcomes for patients across the cancer care continuum.

## Simple Summary

Men and women differ in how cancers develop, respond to treatment, and cause side effects, including damage to the heart. However, these differences are not consistently considered in research or clinical practice. This review summarizes current evidence on how biological sex influences cancer outcomes and treatment-related heart complications across both solid tumors and blood cancers. We aim to highlight the underlying mechanisms, including differences in hormones, genetics, immune responses, and drug metabolism, that may explain these variations. We also discuss how these differences affect treatment safety and effectiveness, particularly the risk of heart damage from cancer therapies. By bringing together evidence from oncology and cardiology, this work supports the need for more personalized, sex-informed approaches in cancer care and cardio-oncology, with the potential to improve patient safety, treatment outcomes, and quality of life.

## 1. Introduction

Sex and gender represent distinct but interacting factors influencing cancer susceptibility, treatment response, and outcomes. Biological sex reflects chromosomal, hormonal, and molecular differences that affect carcinogenesis, pharmacokinetics, immune responses, and cardiovascular (CV) vulnerability, whereas gender encompasses sociocultural and behavioral determinants. Failure to adequately integrate these dimensions introduces bias and limits the translation of research into equitable, precision-based clinical care [1].

Sex differences in cancer susceptibility and prognosis are well-documented and have led to the genesis of the term “sexual dimorphism in cancer” [2]. Males exhibit a higher lifetime probability of developing cancer [3] and worse survival for most non-sex-specific malignancies [4,5]. However, recent epidemiological data indicate increasing cancer incidence in women across several age groups, suggesting that these differences cannot be explained solely by life expectancy [6].

These disparities reflect complex biological and treatment-related mechanisms, including differences in pharmacokinetics, immune regulation, and CV susceptibility. Cardiotoxicity remains a major complication of cancer therapy, with emerging evidence suggesting sex-specific variability in risk and clinical presentation [7,8].

**Figure 1:**
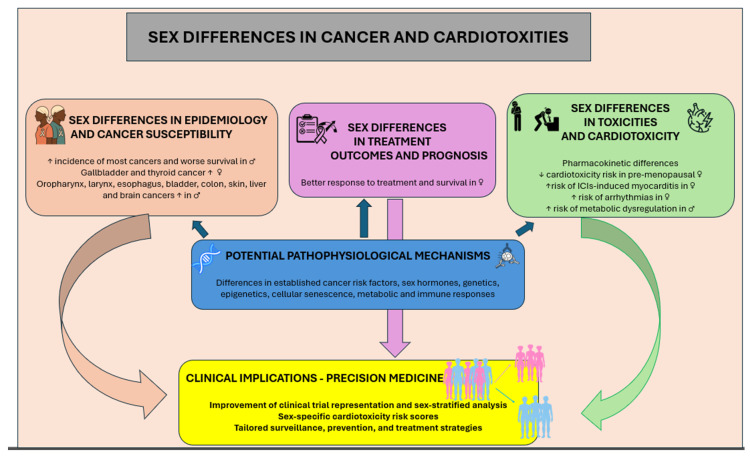
Overview of sex differences in cancer and cardiotoxicities. Conceptual framework illustrating sex-based differences in cancer incidence, treatment outcomes, toxicity patterns, and cardiotoxicity risk, highlighting the underlying biological mechanisms and their clinical implications for precision medicine.

This review aims to synthesize current evidence on sex differences in solid and hematological malignancies, focusing on epidemiology, treatment response, adverse events, and cardiotoxicity (Figure 1). We also explore underlying mechanisms and their clinical implications, emphasizing the need for sex-informed approaches in cardio-oncology. Graphical Abstract maps the sex-specific and intersectional determinants that shape cancer outcomes and cardiotoxicity risk.

## 2. Sex Differences in the Epidemiology and Susceptibility of Solid Tumors and Hematological Malignancies

Sex is an important determinant influencing incidence, rates and patterns of metastasis, treatment response, prognosis and clinical outcomes for many cancers [9]. The most common malignancies are sex-specific—prostate cancer in men and breast cancer in women (though rare in men). Apart from breast cancer, gallbladder and thyroid cancers are more common in females [10]. On the other hand, men have a higher incidence of several types of cancer with lower survival rates, including cancers of the oropharynx, larynx, esophagus, bladder, colon, skin, liver and brain [3,10]. Importantly, sex disparities in cancer incidence are age-dependent and persist across calendar periods, indicating that these differences emerge early in the disease course and cannot be explained solely by treatment exposures, survivorship effects, or differences in life expectancy [11].

### 2.1. Cancer Risk Factors

Sex differences in cancer incidence may be attributed to differences in the prevalence of established cancer risk factors, sex hormones, genetics, epigenetics, cellular senescence, metabolism and immune responses [12]. Sex-related occupational exposures (e.g., pesticides, heavy metals, wood dust, formaldehyde, polycyclic aromatic hydrocarbons, silica dust, diesel exhaust, etc.) are more prevalent among men, largely due to gendered distributions in certain labor sectors such as manufacturing, construction, and agriculture [13]. Behavioral risk factors, such as unhealthy diet [14], tobacco use [15] and alcohol consumption [16] have also been reported more frequently in men, contributing to increased cancer risk.

### 2.2. The Role of Sex Hormones in Cancer Risk

Sex hormones may also have a critical impact on carcinogenesis through several mechanisms that affect cancer stem cell self-renewal, the tumor microenvironment (TME), immune system, and metabolism [2]. Circulating sex hormones may affect the genesis and progression of some cancers like prostate and breast cancer, but exert protective or tumorigenic effects in other cancers. For example, high testosterone levels activate androgen receptors in lung epithelial cells, which may drive tumor growth in non-small cell lung cancer [17]. Furthermore, androgens may enhance hepatitis B virus replication contributing to the higher incidence of hepatocellular carcinoma in men, while estrogens appear to have a protective effect [18]. Estrogen on the other hand is considered pro-tumorigenic for thyroid cancer [19] and progesterone for meningiomas [20]. Hormonal contraception is another source of exogenous sex hormones that may influence cancer susceptibility. Long-term use of oral contraceptives has been associated with a reduced risk of endometrial and ovarian cancers [21], with protective effects persisting for years after discontinuation, but may slightly increase the risk of breast and cervical cancers during current or recent use [22]. The balance of these effects depends on duration of use, age at initiation, and individual risk factors, and may contribute to observed sex-specific patterns in cancer incidence.

Multiple epidemiological studies have consistently shown that women are over-represented among never-smoking lung cancer cases, constituting approximately 2.5-fold higher risk compared to never-smoking men [23], particularly in East Asian populations where up to 70% of female lung cancer patients are never-smokers [24]. Further research indicates associations with hormonal factors, such as higher expression of estrogen receptor α (ERα) in lung tumors of never-smoking women and associations between reproductive timing and lung cancer risk. Early menarche or late menopause have been linked to approximately double the risk, and ERα overexpression frequently co-occurs with EGFR mutations in never-smoking women, suggesting estrogen-driven proliferation and cross-talk with oncogenic pathways in lung carcinogenesis [25].

With advancing age, cancer incidence and prevalence rates increase more sharply in males compared to females, which may be partially explained by sex-based variations in cellular senescence. Estrogens have been shown to enhance the expression of tumor suppressor genes and promote senescence, thereby reducing cancer risk. This hormonal effect may partly explain the lower incidence of certain cancers in premenopausal women compared to men. However, for the majority of cancer types, the extent of sex differences in incidence and prognosis does not correspond to the age-related variations in circulating sex hormone levels.

### 2.3. Genetic Factors in Cancer Risk

Other factors including genetics and epigenetics that contribute to sexual differentiation, maybe key stakeholders. The tumor suppressor TP53 (p53) gene exhibits sex-specific variations that influence cancer development and progression. Germline polymorphisms, such as TP53 Arg72Pro and MDM2 SNP309, can alter p53 activity and show sex-specific associations with cancer risk and age of onset, particularly through hormone-dependent regulation [10,26]. Somatic mutation patterns in TP53 also differ between males and females for certain cancers. In addition, sex-differences in epigenetic processes such as DNA methylation, histone modifications and X-chromosome inactivation can modulate p53 transcriptional activity, DNA repair capacity, and immune responses. Post-translational modifications of p53, influenced by sex hormones, further shape its ability to respond to DNA damage and control cell proliferation, contributing to observed disparities in tumor behavior between men and women.

### 2.4. Metabolic and Immunologic Factors Related to Cancer Risk

Sex-specific metabolic differences may influence cancer risk through distinct patterns of adiposity, hormonal regulation, and insulin signaling. Men tend to accumulate a higher proportion of visceral adipose tissue, which is metabolically active and associated with chronic inflammation, hyperinsulinemia, and increased insulin-like growth factor (IGF-1) signaling—pathways broadly implicated in metabolically driven carcinogenesis. In contrast, premenopausal women preferentially store subcutaneous fat and exhibit greater insulin sensitivity, partly mediated by estrogen, which may confer relative protection against certain cancers. Sex differences in adipokine profiles (e.g., leptin and adiponectin) and inflammatory signaling further modulate cellular proliferation, apoptosis, and the tumor microenvironment, providing biologically plausible mechanisms linking metabolic dimorphism to differential cancer susceptibility [10]. Finally, differences in immune system regulation between males and females can impact cancer risk and outcomes. Overall, females tend to have stronger immune responses than males, with variations in both innate and adaptive immunity observed throughout life [27].

## 3. Sex Differences in Response to Therapies and Outcomes for Solid Cancers

There is growing evidence that sex influences the response to different cancer therapies [28]. Sex hormones have different effects on the TME, affecting the function of cancer-associated fibroblasts, remodeling of the extracellular matrix, angiogenesis and possibly lymphangiogenesis [29]. Strong sex-related differences in the TME have been noted in relation to the profile of infiltrating immune cells, immune checkpoint gene expression and functional pathways, all of which may affect the impact of targeted cancer therapies.

Although sex disparities in cancer progression and prognosis have been documented in several cancer types, relatively little is known about the impact of sex on clinical disease management [30]. Colorectal cancer represents a paradigmatic example of how sex and gender perspectives can meaningfully modify clinical interpretation. Sex-based differences have been described in tumor biology, molecular profiles, treatment tolerance, toxicity patterns, and outcomes, yet sex-stratified analyses remain inconsistently reported in clinical trials and real-world studies. Failure to account for these differences risks obscuring clinically relevant heterogeneity in treatment response and adverse events, underscoring the need to embed sex-informed analyses into trial design and therapeutic decision-making for solid tumors [31].

Intrinsic sex-based differences in anatomy, physiology, body weight and composition (lean and fat body mass), plasma volume, gastric emptying time, plasma protein levels, cytochrome P450 activity, drug transporter function and excretion activity, have a great impact on pharmacokinetic variability in individuals [32]. A recent systematic review assessing the magnitude of sex differences in pharmacokinetics of anticancer agents revealed significant differences (≥20% in clearance) in 15 studies and potentially significant differences in another 8 [33].

Women have a larger volume of distribution when receiving lipophilic drugs, whereas men have larger volume of distribution for water-soluble drugs [34]. Men tend to have increased activity of CYP1A2, CYP2D6 and CYP2E1 enzymes, resulting in increased metabolism of the corresponding drug substrates, while women show higher CYP3A4 activity which is integral in metabolizing most cancer drugs [34]. These sex differences are responsible for potentially up to 20% of treatment responses in women [35].

Sex differences in metabolism and immune response may contribute to differential responses to cancer treatment between men and women. Several studies have noted that women are less likely than men to respond to immune checkpoint inhibitors (ICI) therapy in several cancers, including non-small cell lung cancer and melanoma [36,37]. A recent meta-analysis suggests more nuanced differences in response to ICI therapy (often showing higher benefit for men), and may not be significant when PD-L1 expression is taken into account [38]. Furthermore, similar genomic profiles may have a different response to cancer treatment. In patients with melanoma, the presence of *CFH*, *DGKG or PPP6C* mutations are predictive of a better response to ICI therapy in males but not in females [39].

Sex differences in drug metabolism and elimination—encompassing both renal and metabolic clearance [35], as well as gene transcriptional targeting within the tumor [40]—indicate that drug dosages may need to be adjusted according to sex. However, clinical trials are typically not structured to determine the most effective doses for males and females. Further investigation into sex-specific dose modifications is warranted, particularly for targeted therapies and certain ICI administered at fixed doses, as well as for chemotherapy and antibodies that are dosed based on body surface area and weight.

Sex differences in response to treatment may contribute to differences in cancer outcomes. Data from several trials demonstrate that female sex is associated with improved response to treatment, outcome and survival post-treatment [41]. In addition, women are reported to have a higher rates of toxicities, and severe hematological and symptomatic adverse events from multiple treatment modalities including chemotherapy, ICI and targeted therapy with kinase inhibitors [41,42].

Despite these differences, a recent review of population pharmacokinetic studies on anticancer drugs revealed that only 80 out of 256 studies examined sex as a covariate in their analyses [35]. Women are under-represented in cancer clinical trials for many different types of cancer resulting in approval of drugs based on research conducted mainly in men.

Sex differences in radiosensitivity are also well documented. A recent review suggests a small but significant impact on RT outcomes [43]. Luo et al. [44] analyzed the impact of sex through propensity matched-score analysis in Chinese patients treated with definitive RT for esophageal squamous cell carcinoma, showing higher progression free survival and overall survival in women. The authors conclude on a differential radiosensitivity between sexes, though results should be interpreted with caution, since larger analyses suggest that women have better outcomes for both loco-regional and metastatic esophageal cancer also in the absence of RT [45]. The implications of these differences for radiation-associated CV toxicity are discussed in the dedicated cardiotoxicity section below.

In addition, cancer treatment and outcomes may be confounded by socioeconomic factors, health disparities, and structural inequities. Social determinants of health—such as income, education, race/ethnicity, and access to care—can disproportionately affect women and marginalized populations, potentially amplifying observed sex disparities [46].

## 4. Sex Differences in Clonal Hematopoiesis

Clonal hematopoiesis (CH) arises when a hematopoietic stem cell acquires a somatic mutation that confers a competitive advantage, leading to its clonal expansion and resulting in a substantial fraction of mutant blood cells. CH-driving mutations most frequently affect genes involved in epigenetic regulation (e.g., *DNMT3A*, *TET2*, *ASXL1*), DNA damage response (e.g., *TP53*, *PPM1D*), RNA splicing (e.g., *SRSF2*, *SF3B1*), and intracellular signaling (e.g., *JAK2*). These mutations are strongly linked to hematological malignancies and are often viewed as precursors to leukemia [47]. However, CH—particularly when driven by myeloid malignancy-associated mutations—is also associated with an increased risk of CV disease, particularly atherosclerotic conditions [48,49,50]. In cancer patients, CH also increases the risk of therapy-related myeloid neoplasms and cardiotoxicity [51,52], making it an active focus of research in cardio-oncology.

Sex-related differences in CH are an area of growing interest. While the overall prevalence of CH is similar between men and women, the spectrum of mutated genes differs [49,53]. Notably, *SF3B1*, *SRSF2*, and *ASXL1* mutations are more common in men, whereas *DNMT3A* mutations are enriched in women [49,53]. The mechanisms underlying these differences remain unclear but may involve differential environmental exposures or intrinsic effects of biological sex on mutagenesis or mutant clone expansion. Similarly, although the impact of CH on hematological malignancy risk and mortality appears comparable between sexes, specific mutated genes may exert sex-dependent effects [53,54]. For instance, CH driven by DNA damage response mutations has a significantly greater impact on therapy-related myeloid neoplasms in male than female non-Hodgkin lymphoma patients undergoing autologous stem cell transplantation [54]. Further research is needed to elucidate sex-related differences in the regulation and impact of CH on both hematological and non-hematological diseases. Large-scale sequencing studies are essential to define the gene-specific influence of biological sex on clonal dynamics and disease associations. Complementary experimental studies in animal models are also required to dissect the mechanisms driving these differences.

## 5. Sex Differences in Treatment and Outcomes of Hematological Malignancies

There is limited evidence defining the role of sex differences in response and outcome of patients with hematological malignancies. The incidence of hematological malignancies is generally higher in men than women [55], and this holds true for both myeloid and lymphoid malignancies. Sex differences have been mainly studied in the setting of chronic myeloid neoplasms [ranging from myelodysplastic syndrome (MDS) to myeloproliferative neoplasms (MPN)], where a higher incidence of the disease, a more aggressive phenotype, worse overall survival and a higher incidence of progression to acute myeloid leukemia (AML) were shown in men [56,57]. Molecular characteristics, bone marrow microenvironment [58] and regulation of inflammatory signaling [27] differ in men and women and have been implicated as key factors for the growth of malignant myeloid clones either spontaneously or after tissue-damaging cytotoxic therapy.

In MDS, male sex is an independent predictor of worse survival regardless of age, race and subtype [56]. Large multicenter cohorts in MDS show male sex is an independent adverse prognostic factor even after adjusting for Revised International Prognostic Scoring System and age [56,59]. In MDS, men are enriched for high-risk mutations (*ASXL1*, *SRSF2*, *U2AF1*, *ZRSR2*, *RUNX1*, *TET2*, *IDH2*, *DDX41*) and splicing/DNA-methylation pathways, whereas women more often harbor *DNMT3A*, *TP53* mutations and del(5q) or therapy-related MDS [57,59,60]. These consistent patterns have led to the proposal and validation of sex-informed prognostic models in MDS that integrate sex and genomics, improving risk stratification compared with IPSS-R and reclassifying ~40–56% of patients [59].

In MPN, male sex predicts presentation with primary myelofibrosis rather than ET/PV, higher rates of progression to secondary myelofibrosis/AML, and worse survival, independent of age and driver mutation [57]. Men display higher *JAK2 V617F* allele burdens in CD34+ cells, a greater number of additional somatic mutations, and more high-risk mutations; in contrast, progression in women is more tightly linked to *JAK2* allele burden rather than acquisition of non-driver mutations [57]. At variance with MDS, MPN exhibits a higher prevalence in women. These female patients are generally younger at diagnosis, and frequently present with essential thrombocythemia. Women with MPN generally develop more severe constitutional symptoms and have a higher incidence of vascular complications, in particular abdominal deep vein thrombοsis [61].

MDS/MPN overlap syndrome shows a male predominance and a higher number of somatic mutations, especially in high-risk genes (*ASXL1*, *EZH2*, *RUNX1*, *SETBP1*, *NRAS*, *STAG2*), in men, and this higher prevalence of unfavorable genetic features may explain the worse clinical outcome and the higher risk of transformation to acute myeloid leukemia [57,62]. Differences in drug metabolism may also contribute to the worse outcome in men compared to women, since a higher cytidine deaminase activity in men is associated with a faster azacitidine/decitabine clearance and a suboptimal therapeutic effect [63]. Other potential factors, though less clearly understood, include alterations in the primitive cells’ compartment [57] and implication of hormonal receptors.

In AML, women more often have normal karyotype and *FLT3-ITD*, *NPM1*, *DNMT3A* and *WT1* mutations, while men more frequently harbor complex karyotype and *ASXL1*, *SRSF2*, *U2AF1*, *RUNX1*, and *KIT* alterations, resulting in more women in ELN intermediate/favorable and more men in adverse-risk groups [64,65,66,67]. Large cooperative group analyses and population-based cohorts show female sex associates with better overall survival after adjustment for age, cytogenetics, and mutations, though women experience higher rates of severe chemotherapy-related toxicity and longer hospitalizations [66,68].

In multiple myeloma, incidence is higher in men, but in a large lenalidomide-based trial (Myeloma XI; n = 3894), progression-free and overall survival did not differ by sex, despite women having higher frequencies of high-risk cytogenetic lesions t(14;16) and del(17p) and being more often classified as high- or ultra-high-risk [64,69]. This suggests current regimens can mitigate underlying biological disadvantages in women.

In B-cell malignancies sex differences are less well understood and investigated, but emerging data suggest consistent patterns in incidence, pharmacokinetics, treatment response, and toxicity. Large population-based studies show that men have higher incidence of most lymphoma subtypes, including diffuse large B-cell lymphoma (DLBCL) and follicular lymphoma (FL), and a trend toward higher excess mortality, whereas women often experience better relative survival, particularly in younger and premenopausal age groups [70]. In DLBCL and FL treated with rituximab-containing immunochemotherapy, multiple cohorts and a meta-analysis indicate that male sex is an adverse prognostic factor for overall survival and progression-free survival, while female sex is associated with superior outcomes [70,71,72,73].

Pharmacokinetic studies show that elderly men clear rituximab faster than women, resulting in lower serum levels and shorter exposure; elderly females have reduced rituximab clearance and prolonged half-life, which correlates with better survival and suggests that standard 375 mg/m^2^ dosing may be suboptimal for men [74,75]. Higher rituximab doses (500 mg/m^2^) in elderly male DLBCL patients improve progression-free survival without added toxicity and largely abrogate the male survival disadvantage, supporting sex-adapted rituximab dosing [76]. Similar sex- and weight-related effects on rituximab exposure and outcome are reported in FL and other indolent B-cell lymphomas, where older, heavier males treated with rituximab plus chemotherapy have worse outcomes, likely due to faster clearance, whereas schedules with higher or prolonged exposure (e.g., maintenance) appear to mitigate this gap [77,78].

Beyond rituximab, female patients with DLBCL may also benefit from sex-specific chronotherapy, with afternoon R-CHOP improving survival and reducing infectious and neutropenic complications compared with morning administration, an effect not seen in men [79]. On the other hand, women generally experience more hematologic toxicity and dose reductions, including higher rates of febrile neutropenia and infections, yet often retain equal or superior survival, suggesting higher effective dose intensity and/or more favorable biology [76,78,79]. Mechanistically, sex-related differences in immune function, estrogen signaling and gene expression, and epigenetic regulation have been implicated, for example sex- and age-dependent expression programs in DLBCL linked to estrogens, and sex-biased DNA methylation patterns in chronic lymphocytic leukemia that may contribute to higher male risk [80,81].

Finally, sex also appears to influence outcomes with newer immunotherapies: in large B-cell lymphoma treated with CD19 CAR T-cell therapy, women show significantly higher response rates and superior progression-free and overall survival compared to men without increased severe cytokine release syndrome or neurotoxicity, whereas registry data highlight sex-specific differences in CAR-T-related complications such as higher rates of acute kidney injury and cytokine release syndrome in men and more pancytopenia in women [82,83,84]. Collectively, these findings support a broader, biology-based role of sex in B-cell malignancies, extending beyond rituximab pharmacokinetics to incidence, molecular features, immunobiology, and response to chemoimmunotherapy and CAR-T, and argue for systematic sex-stratified analyses and, where appropriate, sex-adapted dosing and scheduling in B-cell lymphoma trials and practice.

## 6. Sex Differences in Cancer Therapy-Related Cardiovascular Toxicities

Cardiovascular toxicities associated with cancer therapies arise from a complex interplay of factors, including baseline CV risk factors, comorbidities, lifestyle, prior exposure to cardiotoxic therapies, systemic disease burden, and multiple biological and demographic determinants, among which sex represents an important, but not isolated, contributor. Across anticancer treatments, women appear to experience treatment-related toxicities more frequently than men. A pooled analysis of phase II and III trials conducted between 1980 and 2019 (excluding sex-specific cancers) demonstrated a significantly higher risk of severe (grade ≥ 3) adverse events in women compared with men, both overall (+34%) and across chemotherapy (+36%), immunotherapy (+49%), and targeted therapies (+25%) [42]. Importantly, women receiving chemotherapy or immunotherapy also exhibited a higher incidence of lower-grade (grades 1–2) adverse events, suggesting that sex-based differences in CV susceptibility may manifest even at subclinical or mild-to-moderate levels of toxicity [42]. These disparities likely reflect differences in pharmacokinetics, pharmacodynamics, drug metabolism, immune activation, cumulative exposure, and treatment adherence.

Sex-related differences in CV toxicity are underpinned by multiple cellular and molecular mechanisms that influence cardiac structure and function. Biological sex modulates key aspects of myocardial physiology—including inflammatory signaling, fibrotic remodeling, calcium handling, electrophysiology, and contractile function—through the combined effects of sex chromosomes, sex hormones, and downstream transcriptional and epigenetic regulation [85,86]. Sex-related differences in immune regulation are also highly relevant, as females generally exhibit stronger innate and adaptive immune responses, which may contribute to increased susceptibility to immune-mediated cardiotoxicity, such as that observed with ICI [27,87,88]. In parallel, sex-specific patterns of myocardial remodeling and fibrosis, partly mediated by hormonal and genetic factors, may influence the development of long-term cardiac dysfunction following exposure to cardiotoxic therapies [88,89]. Differences in autonomic regulation and responsiveness to catecholamines further contribute to variability in cardiac stress responses and electrophysiological properties [90], while alterations in calcium handling and excitation–contraction coupling may affect myocardial contractility and susceptibility to injury [86,91]. Importantly, these mechanisms should be interpreted as an evolving framework rather than definitive causal pathways, as sex-specific mechanistic data in cardio-oncology remain limited.

A further limitation in interpreting sex-specific mechanisms in cardio-oncology relates to the design of preclinical studies. Historically, many experimental studies of cancer therapy-related cardiotoxicity used predominantly or exclusively male animals, even when both sexes were included, sex-stratified analyses were often absent or underpowered [92,93]. This limitation is particularly relevant in cardio-oncology because preclinical models that include both sexes can demonstrate biologically meaningful differences in susceptibility to cardiotoxicity. For example, male mice appear more susceptible to doxorubicin-induced cardiac injury in several experimental models, with greater myocardial injury, cardiomyocyte vacuolization, apoptosis, and remodeling compared with females [94,95]. Conversely, female susceptibility has been reported in specific immune checkpoint inhibitor myocarditis models, where ICI treatment was associated with enhanced myocardial immune injury and impaired endocrine–cardiac protective signaling [88]. Therefore, mechanistic conclusions derived from single-sex or male-predominant models should be interpreted cautiously, and future preclinical studies in cardio-oncology should systematically include both sexes and report sex-disaggregated outcomes.

Building on this mechanistic framework, sex-related differences in cardiotoxicity manifest clinically across a wide spectrum of phenotypes (Table 1), arising from complex interactions among hormonal status, myocardial stress responses, vascular biology, and metabolic profiles [85].

### 6.1. Anthracyclines and Conventional Cytotoxic Agents

Anthracyclines represent the most extensively studied example of sex-dependent cardiotoxicity. In childhood cancer survivors, female sex is consistently associated with a higher risk of early and late cancer therapeutics-related cardiac dysfunction (CTRCD) and HF [111]. In contrast, among adults, premenopausal women appear relatively protected compared with age-matched men [112], whereas postmenopausal women exhibit risk profiles similar to men [8]. This age-dependent pattern supports a cardioprotective role of estrogens, which are absent in childhood and decline after menopause, making the female heart vulnerable to cardiotoxicity.

Importantly, sex-related risk is not uniform across treatment contexts. In Hodgkin lymphoma survivors exposed to combined mediastinal radiotherapy (RT) and doxorubicin, cardiac hospitalization rates have been reported to be higher in males than females, suggesting a sex–treatment interaction that differs from pediatric anthracycline cohorts [112,113]. These observations highlight that sex-related vulnerability depends not only on biological factors but also on the therapeutic context and exposure pattern. Furthermore, differences in cumulative anthracycline exposure across cancer types represent a critical confounder when interpreting sex-related cardiotoxicity risk. “Classical” treatment protocols vary substantially in cumulative dose, with breast cancer regimens—administered predominantly in female populations—typically involving moderate cumulative doses of doxorubicin (commonly in the range of 240–300 mg/m^2^), whereas treatment protocols for lymphoma and sarcoma, which include more sex-balanced or male-predominant populations, may involve higher cumulative exposures depending on disease subtype and treatment intensity. Given the well-established dose-dependent relationship between anthracyclines and cancer therapy-related cardiac dysfunction (CTRCD), these differences may partly explain apparent sex-related variations in cardiotoxicity risk observed across studies. Importantly, failure to account for cumulative dose may lead to overestimation or misinterpretation of sex as an independent risk factor.

These considerations have direct clinical implications for surveillance strategies. Current cardio-oncology guidelines emphasize risk-adapted monitoring based on cumulative anthracycline exposure; however, integrating both treatment intensity and sex-specific susceptibility may further refine risk stratification. Future studies should incorporate dose-adjusted, sex-stratified analyses to better delineate the independent contribution of biological sex to cardiotoxicity risk.

Sex-related differences extend beyond anthracyclines. For alkylating agents, evidence is more limited but notable. In patients receiving thiotepa-containing conditioning regimens prior to hematopoietic stem cell transplantation, female sex has been independently associated with higher incidence of acute cardiomyopathy and HF, suggesting a potential sex-specific vulnerability to high-dose alkylator myocardial injury in transplant settings (see Table 1).

### 6.2. Targeted Therapies and Immune Checkpoint Inhibitors

Targeted therapies further illustrate sex-related heterogeneity in CV toxicity. With VEGF inhibitors such as bevacizumab, women have demonstrated higher rates of grade ≥ 3 hypertension in clinical trial analyses (see Table 1). Given that VEGF pathway inhibition disrupts endothelial homeostasis, nitric oxide signaling, and microvascular integrity, sex differences in vascular biology and hormonal modulation may contribute to this pattern.

Across tyrosine kinase inhibitor (TKI) classes, pharmacovigilance data reveal sex-stratified differences in reported HF, arrhythmias, and hypertension (see Table 1). In particular, ALK inhibitors have shown differential sex-related reporting patterns for cardiotoxicity in adverse event databases (see Table 1). Furthermore, agent-specific analyses of osimertinib have shown that women exhibit higher risk of HF, whereas men demonstrate higher risk of acute myocardial infarction, underscoring heterogeneity within the TKI class (see Table 1). These findings emphasize the need for systematic sex-stratified safety analyses in modern targeted therapy trials.

For immune checkpoint inhibitors (ICI), myocarditis represents the most concerning cardiac immune-related adverse event. Emerging experimental data support the presence of sex-dependent differences in ICI–associated myocarditis. In a recent preclinical and translational study [88], female mice demonstrated increased susceptibility to ICI-induced myocarditis, characterized by enhanced myocardial immune infiltration, increased T-cell activation, and more pronounced cardiac dysfunction compared with males. These findings are biologically plausible given well-established sex differences in immune regulation, with females generally exhibiting more robust innate and adaptive immune responses. Complementary pharmacovigilance and clinical studies suggest heterogeneous sex-related patterns in ICI cardiotoxicity, with some datasets indicating higher reporting rates in women, although these findings are not consistent across all populations (see Table 1). Mechanistically, sex-related differences in immune activation, T-cell response, and inflammatory signaling may influence susceptibility and clinical phenotype of ICI-associated myocarditis (see Table 1). Taken together, these data support a sex-modulated immune mechanism underlying ICI-associated myocarditis and highlight the need for systematic sex-stratified analyses in both preclinical and clinical studies. Beyond myocardial inflammation, emerging data suggest that ICI may accelerate atherosclerotic plaque progression and vascular events in a sex-related manner, with women demonstrating distinct patterns of plaque evolution and CV risk (see Table 1).

After hematopoietic stem cell transplantation, some studies suggest higher CTRCD incidence in women, although findings are not entirely consistent [114].

### 6.3. Radiotherapy

Radiotherapy (RT) remains a cornerstone of contemporary cancer care, with approximately half of patients receiving RT during their disease course. Thoracic RT can produce a spectrum of radiation-associated CV disease, including coronary, valvular, pericardial, and myocardial injury, and risk is strongly influenced by cardiac dose and treatment technique [115]. Modern paradigms (e.g., involved-site RT for lymphoma; IMRT/VMAT, deep-inspiration breath-hold, and proton therapy in breast cancer) aim to reduce mean heart dose and substructure exposure [115].

Preclinical models support the presence of sex-dependent variability in radiation-associated cardiac injury, particularly in the context of combined treatment modalities. For example, Chmielewski-Stivers et al. demonstrated differential cardiac toxicity following systemic paclitaxel and localized cardiac irradiation, with sex-specific differences in inflammatory response and tissue remodeling, highlighting the potential interaction between chemotherapy, radiotherapy, and biological sex [116].

Although sex-stratified data are limited, survivorship analyses in mediastinal RT cohorts—particularly among Hodgkin lymphoma patients—suggest higher RT-associated CV mortality in women (see Table 1). However, treatment-related factors may modify this risk. In young women, efforts to minimize breast exposure to reduce the risk of RT-induced breast cancer may inadvertently increase cardiac exposure compared with men [117]. Conversely, in men treated with RT for breast cancer, smaller target volumes may result in lower cardiac exposure compared to women, though heart doses are generally low with modern RT [118]. A recent trial assessing sex differences in RT-induced cardiomyopathy in patients hospitalized for acute HF, demonstrated that female sex is associated with lower incidence of atrial fibrillation, hypertension and diabetes, higher rates of pulmonary hypertension and lower in-hospital mortality (see Table 1). Collectively, these data indicate that sex influences not only long-term vascular injury but also myocardial remodeling and HF phenotype after RT.

## 7. Cross-Cutting Cardiotoxic Phenotypes

⮚ *Arrhythmias and QT Prolongation*

Arrhythmia risk is also influenced by sex hormones, particularly by estrogen and testosterone influencing QT interval and susceptibility to channelopathies [119]. Testosterone has a protective effect, shortening QT interval [91]. In line with observation from puberty to menopause, women have longer QT with a higher risk of Torsades de Pointes (TdP). Exogenous hormonal therapies, such as selective estrogen receptor modulators can significantly alter the clinical penetrance of cardiac arrhythmias, prolonging the QT interval by interacting with cardiac ion channels [120]. Women are more prone than men to prolongation of QT interval and TdP induced by medications. Furthermore, this susceptibility to QT prolongation fluctuates during menstrual phases [120]. Conversely, androgen deprivation therapy and enzalutamide increase the risk of QT prolongation and TdP in men [91].

⮚ *Takotsubo syndrome*

Takotsubo syndrome represents a prototypical example of sex-specific vulnerability to cardiac injury, with approximately 80–90% of cases occurring in women, particularly in the postmenopausal period [121]. In the oncology setting, Takotsubo syndrome may present either as primary (triggered predominantly by emotional stress related to cancer diagnosis) or secondary (associated with physical stressors such as cancer therapies, systemic illness, or hospitalization) [122].

Importantly, emerging data suggest that sex-related differences may vary across these phenotypes. In primary Takotsubo, the classical female predominance remains pronounced and is largely attributed to sex-related differences in neurohormonal and autonomic responses, including heightened catecholamine sensitivity and reduced estrogen-mediated cardioprotection. In contrast, in secondary Takotsubo—more frequently encountered in cancer patients—this sex predominance may be attenuated, reflecting the overriding impact of systemic stressors, treatment-related toxicity, and critical illness [122,123].

These observations suggest that while biological sex remains a key determinant of susceptibility, the relative contribution of sex-related mechanisms may be modulated by the clinical context, particularly in cancer patients where treatment-related and disease-related stressors play a central role. This distinction has important implications for risk stratification and highlights the need to consider both sex and triggering context when evaluating Takotsubo cardiomyopathy in cardio-oncology populations.

## 8. Intersectional and Reproductive Considerations

Environmental factors such as socioeconomic status—as well as the interplay of race and sex—further complicate our understanding of sex-based cardiotoxicity differences. For instance, lower socioeconomic status (e.g., unemployment) is independently linked to greater LV ejection fraction (LVEF) decline following doxorubicin in breast cancer survivors [124]. Moreover, within female populations, race appears to further stratify cardiotoxic risk. Black patients with breast cancer develop CTRCD more frequently during doxorubicin treatment, although they have higher incidence of triple-negative breast cancer which can also be subject to more cardiotoxic cancer therapeutic regimens [125]. Furthermore, black women with HER2+ breast cancer develop cardiac dysfunction more often than white patients, even when considering pre-existent CV risk factors such as diabetes or hypertension [46].

Pregnancy represents an additional physiological stressor in cancer survivors previously exposed to cardiotoxic therapy. Furthermore, several studies have shown that women with a history of CTRCD are at significantly increased risk for pregnancy-related CV complications, including HF, arrhythmias, acute coronary syndromes, thromboembolic events, pre-eclampsia, and preterm delivery [126,127]. A systematic review reported left ventricular dysfunction and/or HF during or within one year after pregnancy in up to 28.4% among survivors with prior CTRCD, compared to just 0.24% in those without such history [126]. These findings highlight the importance of pre-conception CV assessment and multidisciplinary care planning in this high-risk population.

## 9. Patient-Reported Outcomes (PROs) and Sex Differences in Cardiotoxicity Assessment

Beyond objective imaging and biomarker-based definitions of cardiotoxicity, patient-reported outcomes (PROs) have emerged as a critical component of contemporary safety assessment in cardio-oncology. Patient-reported outcomes (PROs) capture symptoms, functional status and health-related quality of life from the patient perspective and complement traditional clinician-reported and imaging-based endpoints in CV care [128]. The 2022 ESC Guidelines on cardio-oncology emphasize systematic assessment of CV symptoms and functional impact across the cancer care continuum, supporting a broader safety evaluation beyond left ventricular ejection fraction alone [129]. In heart failure populations, women consistently report worse health-related quality of life and greater symptom burden than men when assessed with validated instruments such as the Kansas City Cardiomyopathy Questionnaire (KCCQ) [130]. In cardio-oncology cohorts—particularly in breast cancer where the treated population is predominantly female—patient-reported symptoms such as fatigue can track with or even precede objective CV decline during potentially cardiotoxic therapy [131]. Qualitative and patient-centered studies in CTRCD highlight that dyspnea and fatigue meaningfully affect daily life and quality of life, reinforcing the value of incorporating PROs into cardiotoxicity assessment and follow-up [132].

## 10. Under-Representation of Women in Cardiovascular Clinical Trials

An important limitation in interpreting sex-specific differences in CV outcomes and cardiotoxicity relates to disparities in clinical trial participation. Historically, women have been under-represented in CV clinical trials, including major HF studies, where female participation has often remained below 30% despite a substantial disease burden in women [133]. This imbalance has important implications for the generalizability of trial findings and may contribute to gaps in sex-specific evidence regarding treatment efficacy, safety, and optimal dosing strategies.

Although recent efforts have aimed to improve sex representation, contemporary analyses suggest that disparities persist across CV and cardio-oncology research. Recent large-scale evaluations continue to demonstrate suboptimal inclusion of women in clinical trials, particularly in studies informing therapeutic decision-making and risk stratification [134,135]. Furthermore, real-world and registry-based analyses indicate that sex-related differences in treatment exposure, outcomes, and adverse event reporting may be incompletely captured due to these participation imbalances [136].

In the context of cardio-oncology, where both cancer biology and CV risk are influenced by sex, under-representation of women in clinical trials may further limit the ability to accurately characterize sex-specific cardiotoxicity profiles and to develop tailored surveillance and management strategies. Addressing these gaps requires systematic inclusion of sex as a biological variable in trial design, as well as consistent reporting of sex-disaggregated outcomes.

## 11. Strategies for Early Identification of Sex-Specific Cancer Treatment-Related Cardiovascular Toxicities

Emerging evidence underscores the importance of sex in influencing the susceptibility, manifestation, and outcomes of CV toxicities associated with cancer therapies [7]. Therefore, developing sex-specific strategies for early identification and prevention is essential to optimize cancer care and mitigate adverse CV effects.

1. **Circulating Biomarkers: Cardiac troponins and natriuretic peptides are used to** detect subclinical myocardial injury in cancer patients [129,137]. In the general population, natriuretic peptide concentrations are approximately two-fold higher in women than in men [138]. By contrast, baseline high-sensitivity cardiac troponin concentrations are typically lower in women than in men and increments in troponin are stronger predictors of CV events in women [139]. On the other hand, in the BiomarCaRE Consortium, NT-proBNP was more strongly associated with incident HF in men than in women (HR per SD 1.89 [1.75–2.05] vs. 1.54 [1.37–1.74]) [81]. Further exploration of these sex-specific biomarkers in cancer patients may offer enhanced sensitivity and specificity for early detection of cardiotoxicity associated with cancer therapies.
2. **Imaging Biomarkers:** While the parameters for LVEF, remodeling patterns and global longitudinal strain (GLS) are the same in men and women, there are clear sex differences in LV mass, size, linear dimensions and volumes based on echocardiographic and cardiac magnetic resonance imaging (cMRI) modalities [140]. Routine echocardiograms with sex-specific reference ranges can be used to detect early changes in LVEF and other cardiac parameters, with GLS identifying subtle myocardial dysfunction before overt cardiotoxicity manifests [141]. Specifically, there may be certain advantages to specific imaging modalities such as cMRI, Positron Emission Tomography and cardiac computed tomography in women compared with men [142].
3. **Genetics:** Sex-informed medicine encompasses genetics, hormones, and immune function, significantly impacting CV disease and cancer. Biological differences, such as X and Y chromosome effects and variations in sex hormones, contribute to differing susceptibilities and disease manifestations in men and women [143]. Emerging pharmacogenetic and genomic data supports the role of Single Nucleotide Polymorphisms (SNPs) in modulating cardiotoxicity risk. For instance, *HAS3 rs2232228* is associated with a dose-dependent risk of anthracycline-induced cardiomyopathy (AA genotype ~8.9× higher risk compared to GG) due to reduced cardiac antioxidant capacity [144]. However, sex-specific effects of these polymorphisms remain mostly unexplored, pointing to a key knowledge gap—particularly given known sex-based differences in vulnerability to therapy-related cardiac injury.

## 12. Clinical Recommendations for Management of Cancer Therapy Related Cardiovascular Toxicity and Sex Differences

Whereas treatment of cardiotoxicity in cancer patients should follow current cardio-oncology and cardiology guidelines, growing evidence indicates that sex-specific differences influence susceptibility, presentation, and outcomes of CV toxicity across cancer therapies [7]. Despite this expanding body of data, sex and gender considerations remain insufficiently integrated into clinical trial design, guideline development, and routine cardio-oncology practice. This gap reflects a challenge of implementation rather than a lack of evidence, highlighting the need to move from descriptive recognition of sex differences toward systematic incorporation into risk stratification, surveillance strategies, and therapeutic decision-making [145].

Prior reviews have described sex-based differences in cardiotoxicity from animal models to patients in epidemiologic studies [8,146]. Pharmacologic differences have also been studied in CV treatments. For instance, with beta blockers, the serum concentration was found to be up to 50% higher in women compared to men. In angiotensin-converting enzyme (ACE) inhibitors, cough was two-fold more likely to occur in women compared to men. In antiplatelet agents, increased bleeding risk was observed in women. Hypokalemia was more common in women treated with thiazide diuretics. In calcium channel blockers peripheral edema occurred more commonly in women. However, while the effects of neurohormonal treatment strategies on their cardioprotective effects have demonstrated significant heterogeneity and a clinically insignificant benefit overall [147], their sex-specific effects have not been carefully studied.

## 13. Prevention of Sex-Specific Cancer Treatment-Related Cardiovascular Toxicities

Sex may have some influence on typical preventive measures which may in turn influence response to treatment and outcomes. For example, variations in hormone levels across menstrual cycles may influence how women respond to beta blockers and angiotensin-converting enzyme (ACE) inhibitors compared with men [148]. It is therefore necessary to integrate hormonal status into treatment planning, especially in therapies that interact with hormonal pathways. Furthermore, the fact that women exhibit up to 50% higher maximal serum concentrations and area under the curve for beta blockers compared to men, and are up to twice as likely as men to experience cough when prescribed ACE inhibitors, should be taken into account when prescribing these drugs as cardioprotective strategies [7].

It is crucial to control CV risk factors such as hypertension or hyperlipidemia before and during cancer therapy. The use of statins has gained recent attention with variable effects in cardioprotection. The STOP-CA trial demonstrated decreased risk of cardiotoxicity with the use of high intensity statins in patients with lymphoma [149], while the PREVENT trial—with a lower median cumulative dose of anthracycline exposure compared to STOP-CA—did not demonstrate significant benefit in breast cancer patients with a similar dose of atorvastatin [150]. On the other hand, a meta-analysis by Petretta et al. reported a significant reduction in coronary events among men, but not among women [151]. Similarly, a large individual patient-level meta-analysis by the Cholesterol Treatment Trialists’ Collaboration suggested a smaller relative risk reduction in major CV events in women than in men, although both sexes benefited overall [152]. In contrast, other studies such as the JUPITER trial demonstrated comparable benefit of statins across sexes [153]. These mixed findings have not been adequately explored in the context of CTRCD, highlighting the need for future cardio-oncology trials to investigate potential sex-based differences in statin response.

Concerning cancer therapy, women are known to have higher risk of adverse drug reactions compared with men, which could lead to drug discontinuation and poorer cancer outcomes [154]. As such, care should be taken to implement sex-specific dosing guidelines to prevent exposure-related cardiotoxicities while not compromising therapeutic efficacy.

In the era of advanced technology and personalized medicine, genetic information is being used to personalize cancer therapy and can be employed to select agents with lower cardiotoxic potential for individuals with high-risk genetic profiles, particularly in women. Leveraging comprehensive biological data from fields like genomics, proteomics, metabolomics, and transcriptomics can help identify sex-specific biomarkers and mechanisms. The multi-omics would not only enhance early detection and diagnosis but would also aid in developing targeted therapies that address the unique biological factors influencing heart disease in each sex, ultimately improving CV outcomes for both women and men [155]. Emerging digital tools, including AI-driven risk stratification models and decision support systems, may further enhance the identification of sex-specific CV risk patterns and optimize personalized prevention strategies in cardio-oncology.

Other overall preventive strategies including CV health promotion such as diet, exercise, smoking cessation, and management of CVRF, and comorbidities such as hypertension, diabetes and dyslipidemia, should employ sex-sensitized approaches to address sex-specific needs and risks.

## 14. Conclusions

Sex differences shape cancer biology, treatment response, and CV outcomes. Men are more vulnerable to non-sex-specific cancers, whereas women more often experience treatment-related toxicities, including cardiotoxicity, reflecting differences in pharmacokinetics, immune responses, hormonal profiles, and genetic susceptibility. While the evidence base supporting sex-informed oncology and cardio-oncology continues to expand, its translation into clinical guidelines and routine practice remains limited. Future research should prioritize sex-stratified trial design, identification of sex-specific biomarkers and risk scores, and evaluation of how sex intersects with age, race, and socioeconomic factors. Integrating sex- and gender-informed approaches is essential to advance precision cardio-oncology and achieve more equitable and effective cancer care.

## Figures and Tables

**Table 1:** Anticancer treatments with reported sex-related differences in cardiotoxicities.

Antineoplastic Therapy	Sex-Related Cardiotoxicities
**Anthracyclines**	- Female sex is an independent risk factor for anthracycline-related cardiac dysfunction/HF in childhood cancer survivors (early clinical cardiotoxicity and late cardiac abnormalities) [96,97,98] - In Hodgkin lymphoma cohorts receiving mediastinal RT plus doxorubicin, cardiac hospitalization incidence is higher in males than females [99]
**Alkylating agents, e.g., thiotepa in high-dose conditioning** **regimens**	- Female sex associated with increased risk of acute cardiomyopathy in high-dose thiotepa-containing conditioning regimens [100]
**VEGF inhibitor** **e.g., bevacizumab**	- Women treated with bevacizumab demonstrated higher rates of grade ≥ 3 hypertension [101]
**TKI, e.g., sunitinib**	- Sex-related differences in CV adverse event reporting observed across TKI classes (higher reported odds for HF in women, signals for arrhythmias stronger in men, hypertension signals varied by sex depending on agent) [102] - ALK inhibitors demonstrate sex-related cardiotoxicity reporting patterns in FAERS (women higher reporting odds for HF and men for certain arrhythmias) [103] - With osimertinib, women showed higher risk of HF, whereas men demonstrated higher risk of acute myocardial infarction [104]
**ICI**	- Pharmacovigilance analyses suggest higher reporting odds of myocarditis in women, although findings are heterogeneous across datasets [105,106] - Sex-related differences in immune activation and inflammatory signaling may influence susceptibility and clinical phenotype of ICI-associated myocarditis [87] - ICI have been associated with accelerated atherosclerotic events and plaque progression, with distinct risk patterns observed in women [107]
**Radiotherapy**	- Higher relative CV mortality reported in women following mediastinal RT for Hodgkin lymphoma [108] - Sex-dependent radiosensitivity and differential dose-volume effects may contribute to long-term CV risk after RT [109] - Sex differences in survival after definitive thoracic RT, suggest possible differences in treatment response and radiosensitivity [44,45] - Sex-related variation in clinical presentation and outcomes of RT-induced cardiomyopathy [110]

## Data Availability

No new data were created or analyzed in this study. Data sharing is not applicable to this article.

## Abbreviations

The following abbreviations are used in this manuscript:

ALK	anaplastic lymphoma kinase
AML	acute myeloid leukemia
CH	clonal hematopoiesis
cMRI	cardiac magnetic resonance imaging
CTRCD	cancer therapeutics-related cardiac dysfunction
CV	cardiovascular
CVD	cardiovascular disease
CVRF	cardiovascular risk factors
EGFR	epidermal growth factor receptor
ERα	estrogen receptor alpha
GLS	global longitudinal strain
HF	heart failure
ICI	immune checkpoint inhibitors
IGF-1	insulin-like growth factor 1
LV	left ventricular
LVEF	left ventricular ejection fraction
MDS	myelodysplastic syndrome
MPN	myeloproliferative neoplasms
NTCP	normal tissue complication probability
PROs	patient-reported outcomes
QT	QT interval
RT	radiotherapy
TdP	Torsades de Pointes
TKI	tyrosine kinase inhibitor
TME	tumor microenvironment
